# Minimally invasive versus open radical resection surgery for hilar cholangiocarcinoma: Comparable outcomes associated with advantages of minimal invasiveness

**DOI:** 10.1371/journal.pone.0248534

**Published:** 2021-03-11

**Authors:** Wei Tang, Jian-Guo Qiu, Xin Deng, Shan-Shan Liu, Luo Cheng, Jia-Rui Liu, Cheng-You Du

**Affiliations:** Department of Hepatobiliary Surgery, The First Affiliated Hospital of Chongqing Medical University, Chongqing, China; Ohio State University Wexner Medical Center Department of Surgery, UNITED STATES

## Abstract

**Background:**

Minimally invasive surgery (MIS) provides a new approach for patients with hilar cholangiocarcinoma (HCCA). However, whether it can achieve similar outcomes to traditional open surgery (OS) remains controversial.

**Methods:**

To assess the safety and feasibility of MIS for HCCA, a systematic review and meta-analysis was performed to compare the outcomes of MIS with OS. Seventeen outcomes were assessed.

**Results:**

Nine studies involving 382 patients were included. MIS was comparable in blood transfusion rate, R0 resection rate, lymph nodes received, overall morbidity, severe morbidity (Clavien–Dindo classification > = 3), bile leakage rate, wound infection rate, intra-abdominal infection rate, days until oral feeding, 1-year overall survival, 2-year overall survival and postoperative mortality with OS. Although operation time was longer (mean difference (MD) = 93.51, 95% confidence interval (CI) = 64.10 to 122.91, P < 0.00001) and hospital cost (MD = 0.68, 95% CI = 0.03 to 1.33, P = 0.04) was higher in MIS, MIS was associated with advantages of minimal invasiveness, that was less blood loss (MD = -81.85, 95% CI = -92.09 to -71.62, P < 0.00001), less postoperative pain (MD = -1.21, 95% CI = -1.63 to -0.79, P < 0.00001), and shorter hospital stay (MD = -4.22, 95% CI = -5.65 to -2.80, P < 0.00001).

**Conclusions:**

The safety and feasibility of MIS for HCCA is acceptable in selected patients. MIS is a remarkable alternative to OS for providing comparable outcomes associated with a benefit of minimal invasiveness and its application should be considered more.

## Introduction

Hilar cholangiocarcinoma (HCCA) (also called Klatskin’s tumor), which originates from the biliary epithelium at the confluence of the right and left hepatic ducts, is the most common malignancy of biliary tracts and is a devastating malignant disease with a poor prognosis [1–2]. Owing to its aggressiveness, late presentation, and refractory nature, the prognosis of patient with unresectable tumor is quite poor with a median survival of less than 1 year. Radical resection for HCCA is considered to be the only potential curative treatment, which can provide possible long-term survival with a 5-year overall survival ranging from 20–40% [3–5]. This tumor always tends to invade the vascular system, the perineural tissue, and major liver parenchyma [6–7]. Therefore, standard radical resection for HCCA includes extrahepatic bile duct resection, major hepatectomy, radical lymphadenectomy, biliary reconstruction, and even vascular resection and reconstruction [8–9]. With the surgical strategy changing from limited bile duct resections to resections including hepatectomy in the past decades, rate of R0 resection and 5-year survival improved a lot [10–12]. However, the complex anatomic features largely increase the difficulty of the surgery, high postoperative morbidity and mortality remain the issues. On the other hand, for the benefits of smaller incision, less intra-operative blood loss, less pain, earlier oral intake, shorter hospital stay, and faster recovery, minimally invasive surgery (MIS), including laparoscopic surgery and robotic surgery, developed rapidly recent years in managing gastrointestinal carcinomas and liver cancers [13]. In the field of liver resection, laparoscopic approach has showed improved short-term outcomes without compromising long-term oncological outcomes compared with open surgery (OS) [14–15]. Thus, laparoscopic hepatectomy has been accepted to be an alternative to conventional open hepatectomy currently [16]. In 2010, the first case of robotic radical resection for hilar cholangiocarcinoma was reported [17]. Robotic system, which features the dexterous EndoWrist^®^ instruments, magnified stereoscopic view, ability of scale motions, and the elimination of surgeon’s physiologic tremors [18–21], offers notable advantages over traditional laparoscope. It enables operators to perform various complicated manoeuvres more stably and precisely than traditional laparoscopic instruments within a limited space and is an innovative MIS approach [22–23]. However, due to the difficulty of oncological resection and biological characteristics of HCCA, MIS for HCCA has been attempted only in limited and highly selected cases worldwide [24–25]. This approach for HCCA is still in its infancy and its efficiency is controversial. Although several studies comparing the clinical outcomes of MIS with OS for HCCA were carried out in the past years, the issues remained for the limited experience in each single center. Therefore, with the aim of comparing the outcomes of MIS with OS and investigate the efficiency of MIS for HCCA further, we systematically summarized the current available data and performed a meta-analysis.

## Materials and methods

### Literature search and study selection

We adhered to the 2009 preferred reporting items for systematic reviews and meta-analysis statement [26]. To provide an adequate overview of the current literature, databases of Medline, Embase, and the Cochrane Library from inception to 18 July 2020 were chosen for screening, an additional search with Google Scholar was performed to supplement the primary search. A combination of the following terms was used as a strategy of literature search: hilar cholangiocarcinoma, perihilar cholangiocarcinoma, Klatskin’s tumor, robotic, laparoscopic, and open (search strategy for Medline is shown in S1 Table). Two authors (Deng and Liu) carried out the search independently and any discrepancies regarding to the study selection were resolved by them. No restriction of language or publication type was set in the search. Title and abstract of each identified publication were screened, and only publications that reported the clinical outcomes of interest were further retrieved. The study protocol was approved by the Science and Research Office of the First Affiliated Hospital of Chongqing Medical University. This study protocol was also registered with the Open Science Framework platform and was available at osf.io/ntmh4.

### Inclusion and exclusion criteria

The inclusion criteria were: (1) published prospective or retrospective cohort studies and randomized controlled trials; (2) studies comparing MIS with OS; (3) studies reported at least 1 of the undermentioned outcomes of interest. The exclusion criteria were: (1) case reports, reviews, letters, editorials and conference reports; (2) studies lacking a control group; (3) studies without available data; (4) studies without a clear description of methods or baseline characteristics.

### Outcomes of interest and definitions

We assessed 17 outcomes of radical resection surgery for hilar cholangiocarcinoma in this meta-analysis, including: (1) Operative parameters: operation time, blood loss, blood transfusion rate, R0 resection rate, and lymph nodes received. (2) Postoperative complications: overall morbidity, severe morbidity (Clavien–Dindo classification > = 3), bile leakage rate, wound infection rate, and intra-abdominal infection rate. (3) Postoperative outcomes: days until oral feeding, length of postoperative analgesia, length of hospital stay, hospital cost, 1-year overall survival, and 2-year overall survival. (4) Postoperative mortality.

Postoperative severe complications were defined according to the Clavien–Dindo classification of surgical complications [27]. Biliary fistula was defined as bile contents in the abdominal drains or leakage found at relaparotomy [28]. Wound infection was diagnosed by the clinical signs (redness, swelling, heat, and pain) of the surgical site and the microbiological analysis of wound samples. Intra-abdominal infection was defined as clinical signs (body temperature higher than 38.5°C combined with leukocyte count higher than 12*10^9^ /L) and the presence of intra-abdominal abscess [29].

### Quality assessment and data extraction

Two reviewers (Deng and Liu) examined the studies independently and extracted data according to the predefined criteria. The extracted data included general information (first author, year of publication, source journal, country, study design, sample size, diagnoses of patients, follow-up period, gender, and the source of clinical data) and 17 outcomes (operation time, blood loss, blood transfusion rate, R0 resection rate, lymph nodes received, overall morbidity, severe morbidity, bile leakage, wound infection, intra-abdominal infection, days until oral feeding, length of postoperative analgesia, length of hospital stay, hospital cost, 1-year overall survival, 2-year overall survival, and postoperative mortality). The obtained data were then compared by the reviewers, inconsistencies were discussed and a third reviewer (Du) was consulted to reach a consensus if necessary. The methodological quality of each included study was assessed using the Newcastle-Ottawa quality assessment scale by Deng and Liu independently [30].

### Statistical analysis

The meta-analysis was carried out in accordance with the Cochrane Reviewer’s Handbook and statistical analyses were performed with the Review Manager Software (Version 5.3, The Cochrane Collaboration, The Nordic Cochrane Centre, Copenhagen, Denmark). The results were presented by odds ratio (OR) or risk difference (RD) with 95% confidence interval (CI) for dichotomous data and mean difference (MD) with 95% CI for continuous data. Heterogeneity among studies was estimated using chi-square test (p < 0.10 represented statistically significant heterogeneity) and I^2^ test (I^2^ > 50% represented statistically significant heterogeneity). When indicating no significant heterogeneity, a fix-effect model was used. Otherwise, a random-effect model was used and a subgroup analysis was performed to explore the discrepancy. Funnel plots were performed to assess the publication bias, and the bias was excluded if a symmetrical distribution was showed. Moreover, a sensitivity analysis was performed by removing each study in turn to evaluate the stability of pooled estimate. Pooled analyses were visualized with forest plots and statistical significance was considered at p < 0.05.

## Results

### Study characteristics and quality assessment

**Fig 1:**
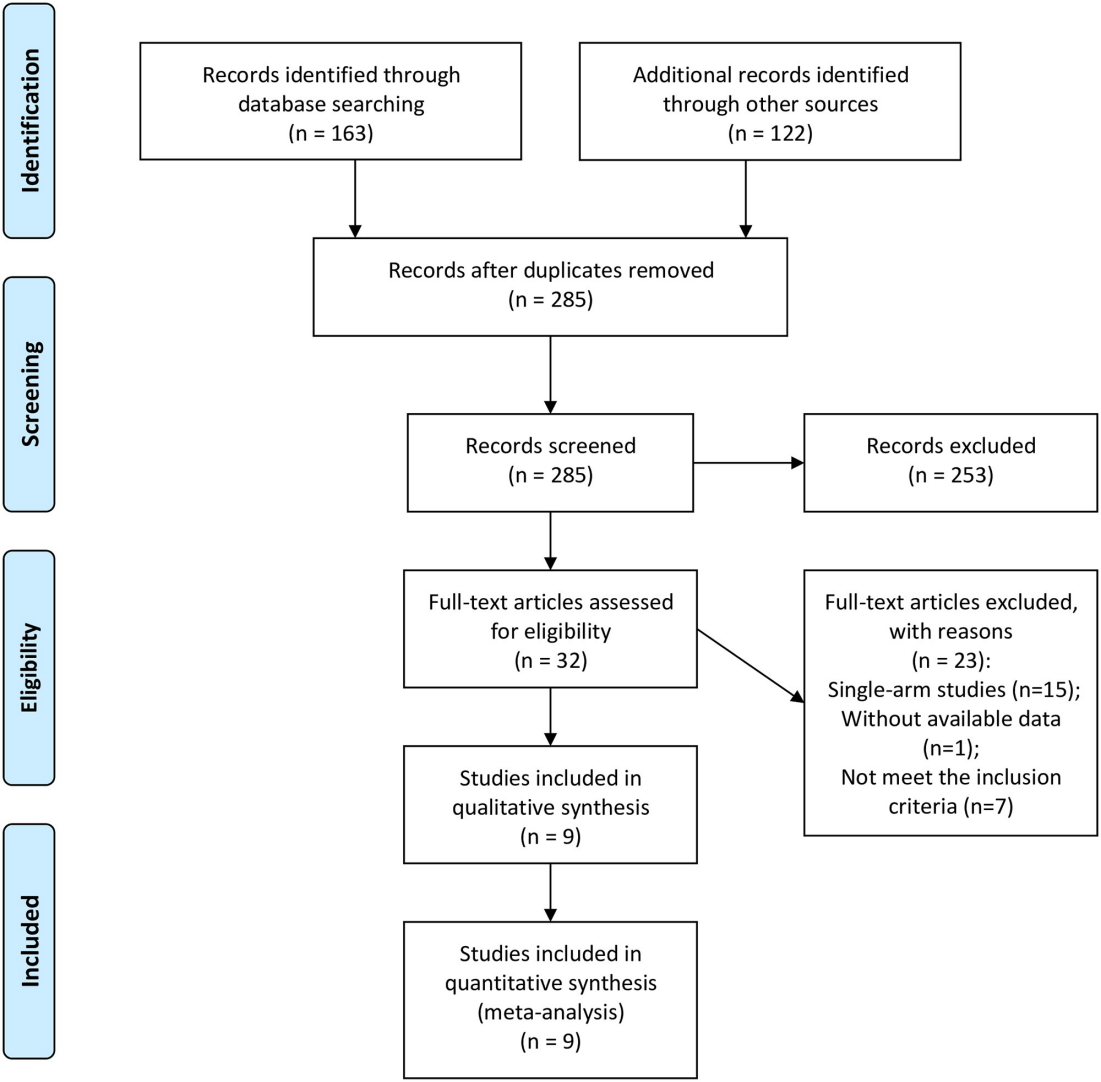
Flow diagram showing process of literature search and study selection.

The PRISMA flow diagram of literature search strategies is shown in Fig 1. Initially, 285 articles were found using combination of search terms and 253 irrelevant articles were excluded according to the inclusion and exclusion criteria after screening titles and abstracts. After assessment of full text, 23 studies were removed for the following reasons: 7 studies failed to meet the inclusion criteria, 15 studies were single-arm studies, available data were lacked in 1 study. Finally, 9 studies (all were retrospective cohorts) were included in the meta-analysis [31–39].

The baseline characteristics of the 9 included studies were summarized in Table 1. All studies were well designed to compare two arms: MIS (robotic or laparoscopic) and OS for HCCA. The studies period ranged from 2014 to 2020. Analyses were performed on 382 patients, of whom 164 (42.9%) underwent MIS and 218 (57.1%) underwent OS. Three studies investigated patients with Bismuth–Corlette type I hilar cholangiocarcinoma, 1 study focused on type IIIb, and 5 studies were about hilar cholangiocarcinoma of all Bismuth–Corlette types. Most of the included studies showed satisfactory quality with selection criteria, comparability of patient characteristics, and adequate follow-up. All cohorts got 7 or more stars as shown in S2 Table.

**Table 1:** Characteristics of included studies.

Reference	Year	Region	Study design	Sample size		Bismuth–Corlette classification	Laparoscopic or robotic surgery	Age: median(range)/mean±SD		Gender: male/female	
				MIS	OS			MIS	OS	MIS	OS
**Xu et al**. [31]	2016	China	Retrospective cohort	10	32	Mixed	Robotic	54(36–77)	59(37–77)	8/2	20/12
**Zhang et al**. [32]	2019	China	Retrospective cohort	14	9	Mixed	Laparoscopic	65.4±8.9	65.4±6.9	7/7	3/6
**Jiang et al**. [33]	2020	China	Retrospective cohort	54	54	Bismuth type I	Laparoscopic	67.7±2.3	67.7±2.2	29/25	28/26
**Zhu et al**. [34]	2018	China	Retrospective cohort	10	24	Bismuth type I	Laparoscopic	62.0±7.4	59.2±8.1	6/4	13/11
**Gong et al**. [35]	2014	China	Retrospective cohort	14	5	Mixed	Laparoscopic	58.5±3.6	54.9±4.5	8/6	3/2
**Chou et al**. [36]	2020	China	Retrospective cohort	16	31	Mixed	Robotic	68.0±7.0	60.0±9.0	11/5	20/11
**Duan et al**. [37]	2019	China	Retrospective cohort	13	14	Bismuth type I	Laparoscopic	59.2±8.0	62.0±7.4	N/A	8/6
**Chai et al**. [38]	2019	China	Retrospective cohort	17	17	Bismuth type IIIb	Laparoscopic	N/A	N/A	N/A	N/A
**Ratti et al**. [39]	2020	Italy	Retrospective cohort	16	32	Mixed	Laparoscopic	61(48–81)	63(43–80)	8/8	17/15

### Meta-analysis of operative parameters

**Fig 2:**
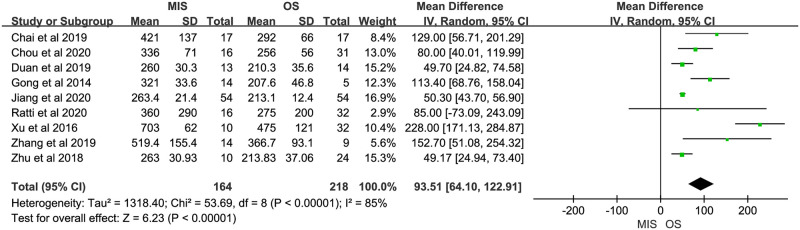
Meta-analysis of studies comparing operation time between minimally invasive surgery and open surgery groups based on a random-effect model.

**Fig 3:**
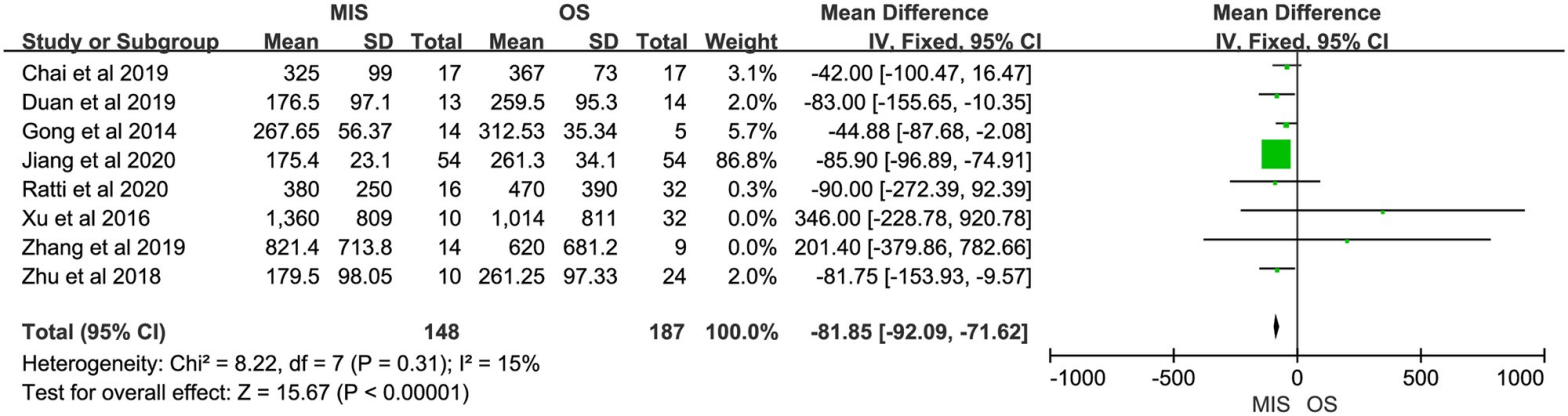
Meta-analysis of studies comparing blood loss between minimally invasive surgery and open surgery groups based on a fixed-effect model.

**Fig 4:**
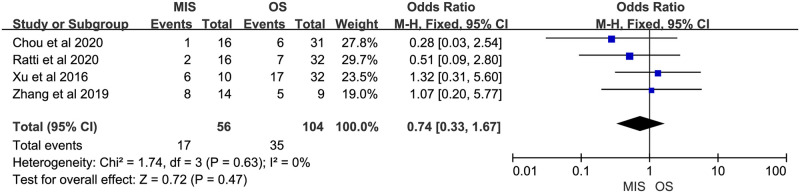
Meta-analysis of studies comparing blood transfusion rate between minimally invasive surgery and open surgery groups based on a fixed-effect model.

**Fig 5:**
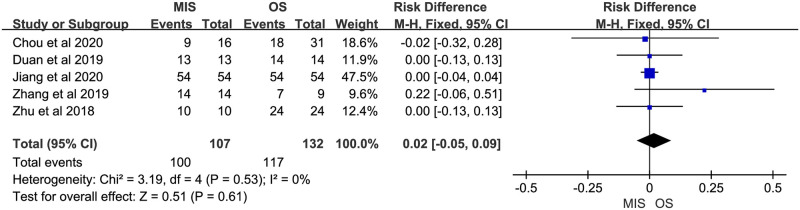
Meta-analysis of studies comparing R0 resection rate between minimally invasive surgery and open surgery groups based on a fixed-effect model.

**Fig 6:**
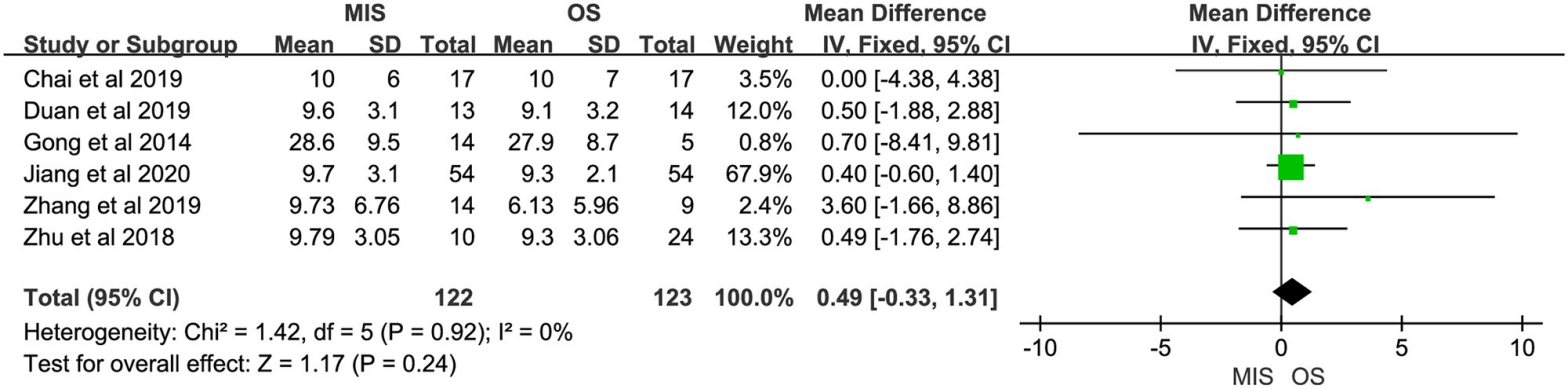
Meta-analysis of studies comparing lymph node received between minimally invasive surgery and open surgery groups based on a fixed-effect model.

Nine studies reported operation time, all of them suggested it was longer in MIS. There was significant heterogeneity (P < 0.00001, I^2^ = 85%). A random-effect model indicated a significant difference between MIS and OS, operation time of MIS was much longer than OS (MD = 93.51, 95% CI = 64.10 to 122.91, P < 0.00001; Fig 2). Eight studies reported blood loss, with 5 of them indicating no significant difference between MIS and OS and 3 indicating less blood loss in MIS. No significant heterogeneity was observed (P = 0.31, I^2^ = 15%). A fixed-effect model was used and pooled results showed blood loss was much less in MIS than OS (MD = -81.85, 95% CI = -92.09 to -71.62, P < 0.00001; Fig 3). Data on blood transfusion rate were available in 4 of the 9 studies. All studies suggested there was no significant difference between MIS and OS. No significant heterogeneity was observed (P = 0.63, I^2^ = 0%). A fixed-effect model showed comparable blood transfusion rate between MIS and OS (OR = 0.74, 95% CI = 0.33 to 1.67, P = 0.47; Fig 4). Five studies compared the R0 resection rate of MIS with OS groups, the results of 5 studies indicated no significant difference between MIS and OS patients, there was no significant heterogeneity in the R0 resection rates of the 5 studies (P = 0.53, I^2^ = 0%), and a fixed-effect model showed that the R0 resection rate of MIS group was similar to the rate of OS group (RD = 0.02, 95% CI = -0.05 to 0.09, P = 0.61; Fig 5). Five studies provided data on the lymph nodes received, with all of them suggesting no significant difference between MIS and OS, no significant heterogeneity among the 5 studies was observed (P = 0.92, I^2^ = 0%), and pooled results of a fixed-effect model were found to be equivalent between MIS and OS for the lymph nodes received (MD = 0.49, 95% CI = -0.33 to 1.31, P = 0.24; Fig 6).

### Meta-analysis of postoperative complications

**Fig 7:**
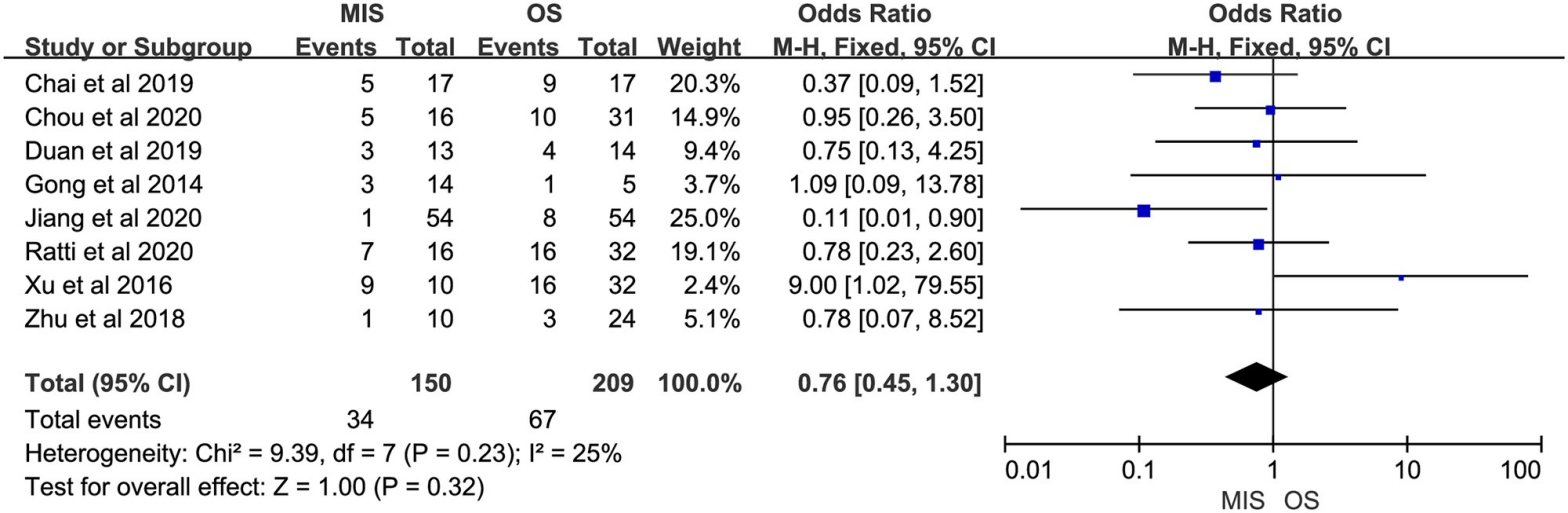
Meta-analysis of studies comparing overall morbidity between minimally invasive surgery and open surgery groups based on a fixed-effect model.

**Fig 8:**
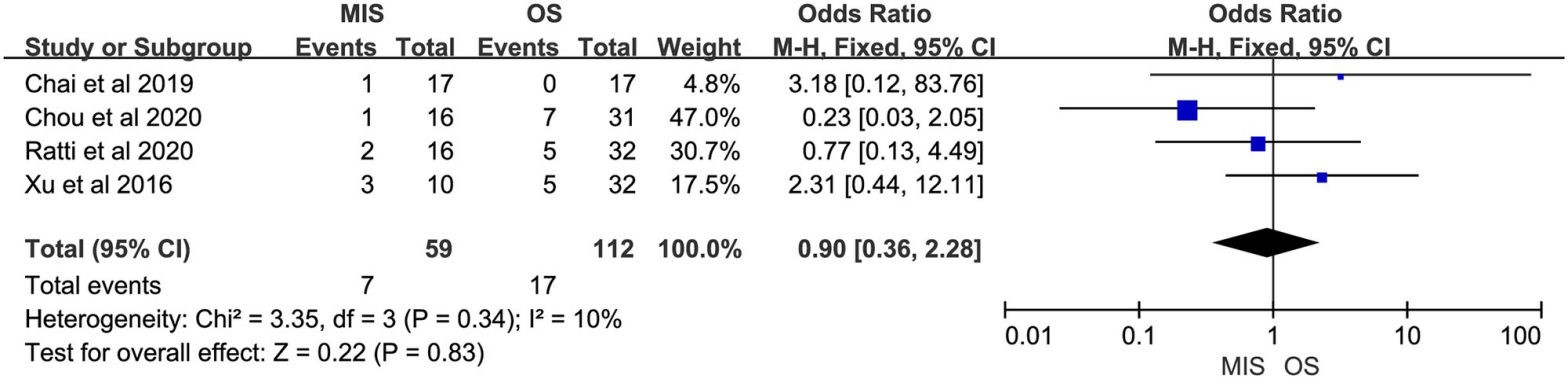
Meta-analysis of studies comparing severe morbidity between minimally invasive surgery and open surgery groups based on a fixed-effect model.

**Fig 9:**
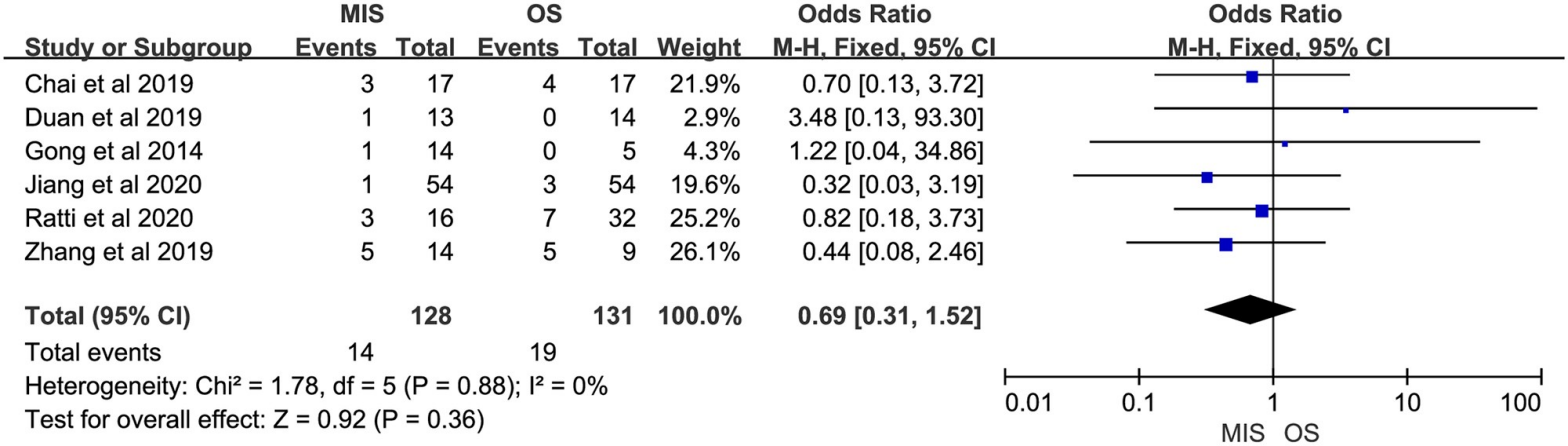
Meta-analysis of studies comparing bile leakage rate between minimally invasive surgery and open surgery groups based on a fixed-effect model.

**Fig 10:**
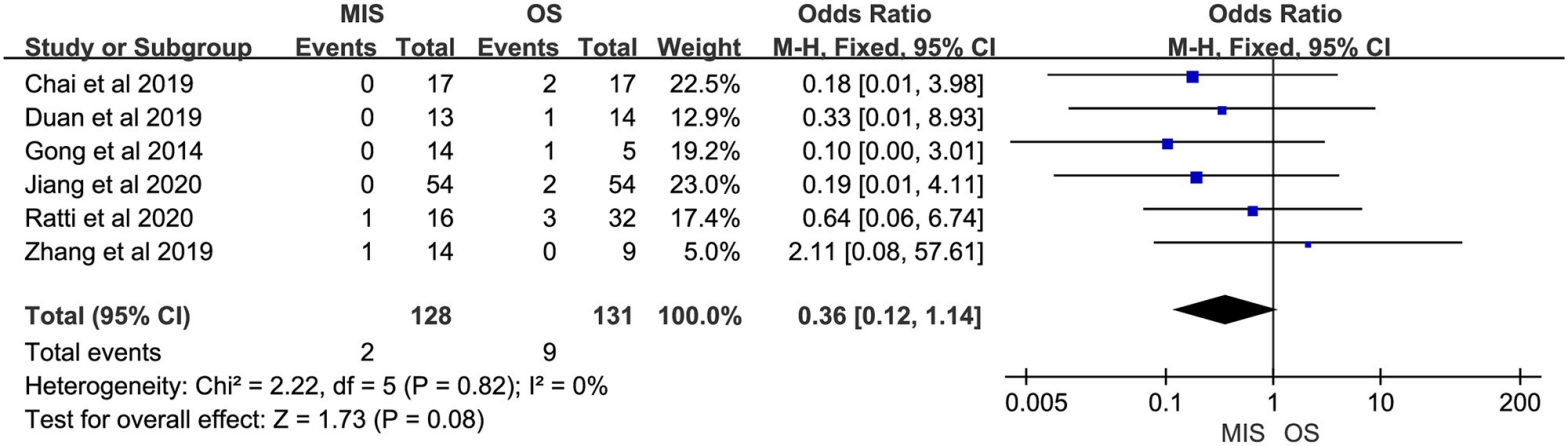
Meta-analysis of studies comparing wound infection rate between minimally invasive surgery and open surgery groups based on a fixed-effect model.

**Fig 11:**
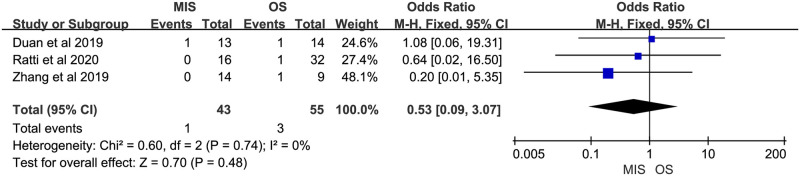
Meta-analysis of studies comparing intra-abdominal infection rate between minimally invasive surgery and open surgery groups based on a fixed-effect model.

Eight studies reported overall morbidity. Seven of them suggested no significant difference between MIS and OS, 1 study showed a significant higher overall morbidity in MIS group. There was no significant heterogeneity (P = 0.23, I^2^ = 25%). A fixed-effect model indicated there was no significant difference between MIS and OS for overall morbidity (OR = 0.76, 95% CI = 0.45 to 1.30, P = 0.32; Fig 7). Four studies provided data on severe morbidity, with all of them suggesting no significant difference between MIS and OS patients. Insignificant heterogeneity among the 4 studies was found (P = 0.34, I^2^ = 10%). A fixed-effect model revealed similar severe morbidity between MIS and OS (OR = 0.90, 95% CI = 0.36 to 2.28, P = 0.83; Fig 8). Six studies reported bile leakage rate, all showed no significant difference existed between MIS and OS groups. A fixed-effect model revealed similar bile leakage rate between MIS and OS (OR = 0.69, 95% CI = 0.31 to 1.52, P = 0.36; Fig 9). There was no significant heterogeneity observed (P = 0.88, I^2^ = 0%). Six studies reported wound infection rate, all studies suggested there was no significant difference between MIS and OS. No significant heterogeneity was observed (P = 0.82, I^2^ = 0%). A fixed-effect model revealed a lower but insignificant wound infection rate in MIS than OS group (OR = 0.36, 95% CI = 0.12 to 1.14, P = 0.08; Fig 10). Three studies reported intra-abdominal infection rate. All suggested no significant difference between MIS and OS. No significant heterogeneity was observed (P = 0.74, I^2^ = 0%). A fixed-effect model revealed comparable intra-abdominal infection rate between MIS and OS group (OR = 0.53, 95% CI = 0.09 to 3.07, P = 0.48; Fig 11).

### Meta-analysis of postoperative outcomes

**Fig 12:**
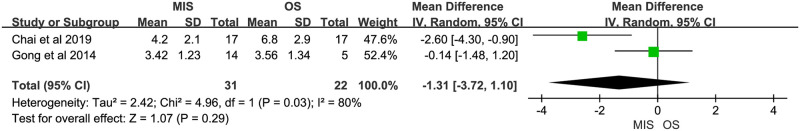
Meta-analysis of studies comparing days until oral feeding between minimally invasive surgery and open surgery groups based on a random-effect model.

**Fig 13:**
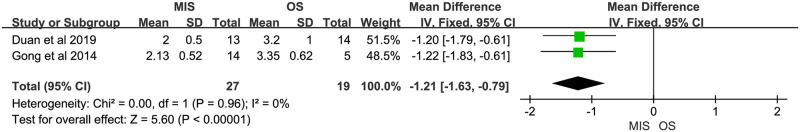
Meta-analysis of studies comparing length of postoperative analgesia between minimally invasive surgery and open surgery groups based on a fixed-effect model.

**Fig 14:**
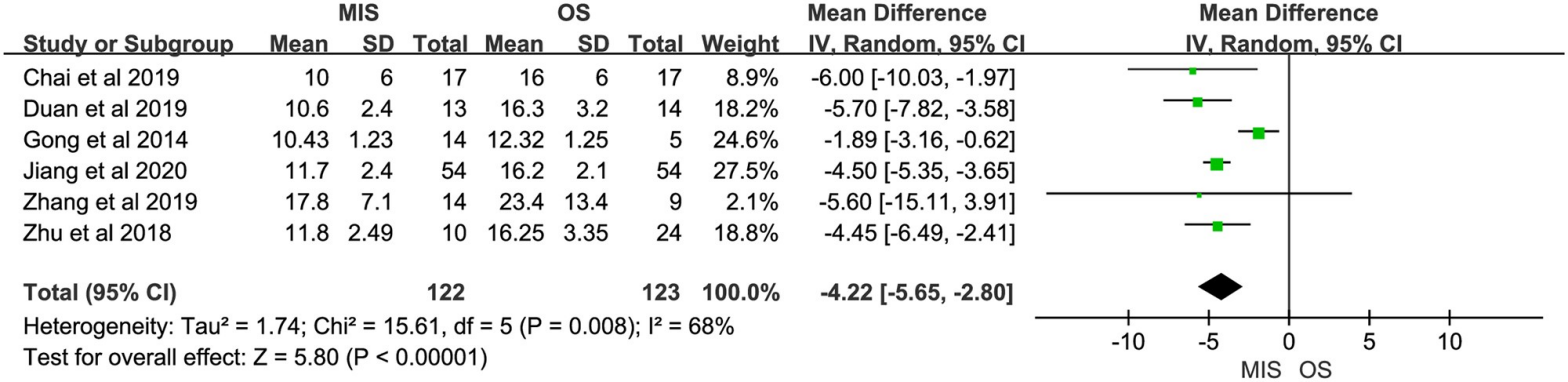
Meta-analysis of studies comparing length of hospital stay between minimally invasive surgery and open surgery groups based on a random-effect model.

**Fig 15:**
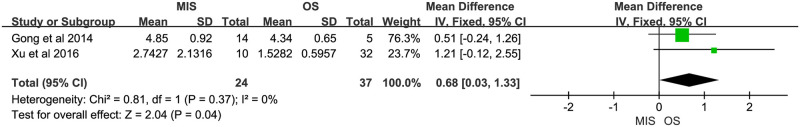
Meta-analysis of studies comparing hospital cost between minimally invasive surgery and open surgery groups based on a fixed-effect model.

**Fig 16:**
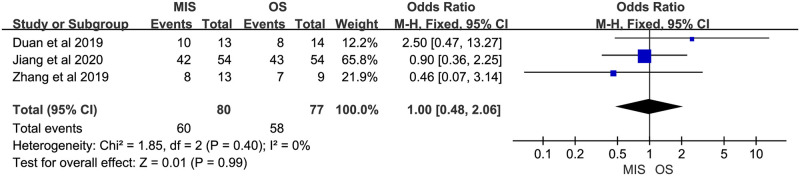
Meta-analysis of studies comparing 1-year overall survival between minimally invasive surgery and open surgery groups based on a fixed-effect model.

**Fig 17:**
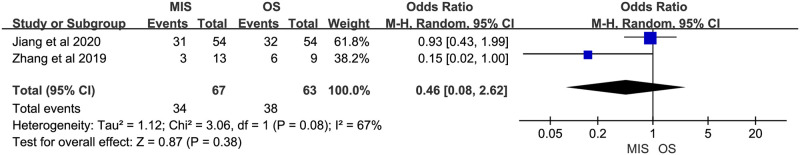
Meta-analysis of studies comparing 2-year overall survival between minimally invasive surgery and open surgery groups based on a random-effect model.

Two studies reported days until oral feeding. One study suggested no significant difference between MIS and OS, another showed a significant less days until oral feeding in MIS group. Significant heterogeneity was observed (P = 0.03, I^2^ = 80%). A random-effect model suggested no significant difference between MIS and OS (MD = -1.31, 95% CI = -3.72 to 1.10, P = 0.29; Fig 12). Two studies reported length of postoperative analgesia. Both indicated a significant shorter length of postoperative analgesia in MIS. No significant heterogeneity was observed (P = 0.96, I^2^ = 0%). A fix-effect model showed a significant shorter length of postoperative analgesia in MIS compared with OS (MD = -1.21, 95% CI = -1.63 to -0.79, P < 0.00001; Fig 13). Six studies reported length of hospital stay. Five of them suggested a significant shorter length of hospital stay in MIS patients, and the rest one showed comparable length of hospital stay between MIS and OS groups. Significant heterogeneity was observed (P = 0.008, I^2^ = 68%). A random-effect model revealed a significantly shorter length of hospital stay in MIS compared with OS (MD = -4.22, 95% CI = -5.65 to -2.80, P < 0.00001; Fig 14). Two studies provided data about hospital cost. Both suggested no significant difference between MIS and OS. There was no significant heterogeneity (P = 0.37, I^2^ = 0%). A fixed-effect model showed significant higher hospital cost in MIS group (MD = 0.68, 95% CI = 0.03 to 1.33, P = 0.04; Fig 15). Three studies reported 1-year overall survival. All suggested there was no significant difference between MIS and OS. No significant heterogeneity was observed (P = 0.40, I^2^ = 0%). A fixed-effect model revealed comparable 1-year overall survival between MIS and OS (OR = 1.00, 95% CI = 0.48 to 2.06, P = 0.99; Fig 16). Two studies reported 2-year overall survival. Both studies suggested comparable 2-year overall survival between MIS and OS. Significant heterogeneity was observed (P = 0.08, I^2^ = 67%). A random-effect model revealed there was no significant difference between MIS and OS groups (OR = 0.46, 95% CI = 0.08 to 2.62, P = 0.38; Fig 17).

### Meta-analysis of postoperative mortality

**Fig 18:**
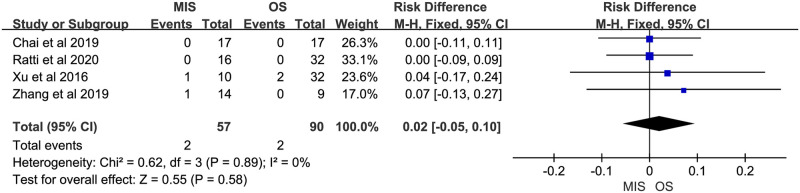
Meta-analysis of studies comparing postoperative mortality between minimally invasive surgery and open surgery groups based on a fixed-effect model.

Four studies reported postoperative mortality, all suggested no significant difference between MIS and OS. No significant heterogeneity was observed (P = 0.89, I^2^ = 0%). A fixed-effect model suggested comparable postoperative mortality between MIS and OS (RD = 0.02, 95% CI = -0.05 to 0.10, P = 0.58; Fig 18).

### Publication bias assessment, sensitivity analysis, and subgroup analysis

There was no evidence of publication bias for blood loss, blood transfusion rate, R0 resection rate, lymph nodes received, overall morbidity, severe morbidity, bile leakage rate, intra-abdominal infection rate, days until oral feeding, length of postoperative analgesia, hospital cost, 1-year overall survival, 2-year overall survival, and postoperative mortality, with a symmetrical appearance on funnel plots. For operation time, wound infection rate, and length of hospital stay, funnel plots showed an asymmetry which suggested negative studies might be less reported. According to the sensitivity analysis, most of overall results did not change after the exclusion of a single study except overall morbidity and wound infection. To investigate the source of heterogeneity among studies, a subgroup analysis was carried out by stratifying the analysis according to several important factors, including: sample sizes, types of Bismuth–Corlette classification, and types of MIS (Table 2). All subgroup results were in line with the main results.

**Table 2:** Subgroup analysis.

Outcomes	Subgroup	Studies (n)	Effect estimate (95% CI)	P value	Heterogeneity	Inconsistency with the overall result
**Operation time**	Sample size < 30	3	93.41 (34.69, 152.14)	P = 0.002	P = 0.01, I^2^ = 77%	
	Sample size > = 30	6	97.43 (56.10, 138.75)	P < 0.00001	P < 0.00001, I^2^ = 88%	
	Bismuth I	3	50.19 (44.02, 56.36)	P < 0.00001	P = 1.00, I^2^ = 0%	
	Mixed	6	133.80 (82.97, 184.63)	P < 0.00001	P = 0.003, I^2^ = 73%	
	Robotic surgery	2	152.56 (7.55, 297.57)	P = 0.04	P < 0.0001, I^2^ = 94%	
	Laparoscopic surgery	7	67.47 (46.81, 88.14)	P < 0.00001	P = 0.01, I^2^ = 62%	
	Overall	9	93.51 (64.10, 122.91)	P < 0.00001	P < 0.00001, I^2^ = 85%	
**length of hospital**	Sample size < 30	3	-3.87 (-7.20, -0.55)	P = 0.02	P = 0.009, I^2^ = 79%	
**stay**	Sample size > = 30	3	-4.55 (-5.32, -3.78)	P < 0.00001	P = 0.77, I^2^ = 0%	
	Bismuth I	3	-4.64 (-5.37, -3.90)	P < 0.00001	P = 0.58, I^2^ = 0%	
	Mixed	3	-3.56 (-6.76, -0.36)	P = 0.03	P = 0.13, I^2^ = 51%	
	Robotic surgery	0	N/A	N/A	N/A	N/A
	Laparoscopic surgery	6	-4.22 (-5.65, -2.80)	P < 0.00001	P = 0.008, I^2^ = 68%	
	Overall	6	-4.22 (-5.65, -2.80)	P < 0.00001	P = 0.008, I^2^ = 68%	

## Discussion

MIS features smaller incision, less pain, faster recovery, and earlier discharge. It has revolutionized the treatment concept of hepatobiliary surgery [40, 41]. Minimally invasive major hepatectomy and pancreatoduodenectomy have been accepted and widely performed by more and more surgeons with no inferiority to OS [42–44]. Furthermore, in the fields of treatment for gallbladder stone, minimally invasive procedure has been even viewed as a standard treatment method over the years. In this circumstances, the idea of MIS for HCCA formed spontaneously. Recently, the adoption of MIS in treating Bismuth type-I to type-IV HCCA increased for the rapid development of surgical methods and equipment and the accumulation of MIS experience [17]. Compared to laparoscopic procedure, robotic techniques provide fine dissection, endoscopic suturing, and microanastomosis better, and most importantly, enable operating in a delicate space [45–47]. With the increased experience in robotic surgery, biliary reconstruction was no longer a contraindication for MIS [48–50]. However, the complexity of portal dissection and biliary reconstruction is still a big concern that restricts the development of minimally invasive approach and most studies were limited in highly selected cases. MIS for HCCA is still in its infancy [51, 52]. The lack of large volume studies and the uncertainty of the clinical outcomes of MIS for HCCA restricted the progress of minimally invasive concept in HCCA severely. Thus, at these early stages, further studies comparing the outcomes of MIS with OS are crucial in expanding the application of minimally invasive method in HCCA. With the aim of assessing the safety and feasibility of laparoscopic surgery and robotic surgery for HCCA, we conducted this meta-analysis.

R0 resection, which means no residual tumor in the resection margins of the bile ducts, is viewed as the most important factor affecting long-term survival in the surgical therapy for HCCA. The positive rate of resection margins of bile ducts directly affected the prognosis of patient and patient without R0 resection was demonstrated to have a dismal survival [53–55]. Thus, to achieve R0 margins is crucial to curative treatment in HCCA resectional surgery [8]. However, R0 resection was considered to be hard to guarantee for the following 3 reasons. First, numerous vital structures around the tumor in the liver hilum. Second, the difficulty in determining the exact length and width of microscopic tumor extension pre-operatively and intra-operatively for the microscopic biological extending nature along the bile ducts [56]. Third, the accuracy, sensitivity, and specificity of intra-operative frozen-section examination of ductal margins were only 56.5%, 75.0%, and 46.7%, respectively [57]. The uncertainty of R0 resection of MIS for HCCA was a big concern. In this meta-analysis, our result indicated that, compared with traditional OS, patients undergoing MIS for HCCA experienced comparable R0 resection rate. This result is quite crucial and encouraging for the further development of MIS.

The main histopathologic type of HCCA was adenocarcinoma and the lymph node metastasis rate ranged from 30.0% to 60.0% [35]. Lymphadenectomy is an essential component of radical resection for HCCA and is another factor affecting long-term survival. However, the scope of lymphadenectomy is controversial. In most cases, the regional lymph nodes ranging from the hepatoduodenal ligament to the superior border of the pancreatic head should be routinely dissected [58, 59]. In our study, the pooled result indicated that there was no statistical difference regarding the lymph nodes received between the two groups, with relatively lower in OS group. Furthermore, it is worth to note here that excessive dissection of lymph nodes around the hepatic artery might result in the mechanical injury of vessel and increase the incidence rate of hepatic artery pseudoaneurysm. In Zhang’s cases [32], one patient suffered from postoperative bleeding, and the reason was confirmed to be the rupture of hepatic artery pseudoaneurysm during reoperation.

Whether postoperative morbidity is more frequent in MIS remains uncertain. Liver failure was one of the most common cause of death after surgery for HCCA [31, 60]. Previous related studies considered pre-operative embolism of the portal vein might increase postoperative residual liver volume to prevent perioperative death due to acute liver failure [61]. However, some other studies believed the functional reserve of liver was more essential in affecting post-hepatectomy liver failure and postoperative mortality than the residual liver volume preserved [62, 63]. In Xu’s experience [31], percutaneous transhepatic biliary drainage should be considered preoperatively in reducing postoperative liver failure and mortality. Bile leakage was thought to be one of the most common complications that might prolong hospital stay following radical resection for HCCA [64]. Improvement of the surgeon’s skill in suturing the bile-duct wall was the key factor in reducing bile leakage rate, while early detection and timely conservative therapy (including abdominal drainage) were essential to shorten hospital stay [60, 64, 65]. Hepatic arterial pseudoaneurysm was an uncommon complication after surgery for HCCA, in robotic surgery, Xu et al. [31] thought an imperceptible mechanical injury might do some damage to the endothelium by the frequent grasping of the vessels due to the lack of tactile feedback from the instrument. And one patient received reoperation for the rupture of hepatic arterial pseudoaneurysm in their cases. Besides, potential risk factors of high morbidity included prolonged operative time and the physical and hemodynamic stress placed by prolonged duration of pneumoperitoneum over 15 mmHg [66]. Our pooled results indicated that the postoperative morbidity and mortality in MIS were comparable with OS for HCCA.

In addition, patients undergoing MIS for HCCA experienced prolonged operative time compared with traditional OS. This was thought to be due to the surgeons’ inexperience in the early phase of learning curve, the technical limitations of minimally invasive approach in liver mobilization and retraction, and the forced use of anterior approach for vascular management [31]. This phenomenon is commonly observed in the development process of a laparoscopic surgery, and the prolonged operative time will gradually improved with the accumulation of MIS experience and the surgical techniques.

However, we have to acknowledge some limitations in our study. First, all studies included in this systematic review were small retrospective cohort studies, no high volume study or well-designed randomized controlled trial was included. Further studies with larger patient cohorts and sufficient follow-up duration are required to demonstrate the results. Second, the existence of significant heterogeneity in some outcomes was not explained well enough by subgroup analysis. Third, definitions of some outcomes were not clear or uniform in different studies. Fourth, under the current circumstances, MIS for HCCA was only performed for highly selected patients at very limited institutions. In this review, most of the included studies were conducted in China, the scarcity of worldwide report might cause some potential bias.

## Conclusions

In conclusion, this is to date the first meta-analysis comparing MIS with OS for HCCA worldwide. Our initial results demonstrated that, in selected patients, MIS was not inferior to OS in consideration of blood transfusion rate, R0 resection rate, lymph nodes received, overall morbidity, severe morbidity, bile leakage rate, wound infection rate, intra-abdominal infection rate, days until oral feeding, patent overall survival, and postoperative mortality. Although the expenditure for MIS was higher due to the instrumental cost, it obtained a benefit of minimal invasiveness, that was less blood loss, less postoperative pain, and shorter hospital stay. The cost-effectiveness of MIS for HCCA is acceptable. Therefore, MIS is a valuable option for patients with HCCA for providing a remarkable alternative to OS, the application of MIS for HCCA should be considered more worldwide.

## Supporting information

S1 Table Search strategy for Medline. (PDF)

S2 Table Newcastle-Ottawa quality assessment scale for cohort studies. (PDF)

S1 Checklist PRISMA-2009-checklist. (PDF)
