# Supplementary material for: Minimally invasive versus open radical resection surgery for hilar cholangiocarcinoma: Comparable outcomes associated with advantages of minimal invasiveness
Source: PLoS One. 2021 Mar 11;16(3):e0248534. doi: 10.1371/journal.pone.0248534 (PMC7951922; doi:10.1371/journal.pone.0248534)
Supplement: S1 Table — (PDF) [file pone.0248534.s001.pdf]

#1	(hilar cholangiocarcinoma) OR (perihilar cholangiocarcinoma) OR (klatskin's tumour)
#2	robotic OR laparoscopic OR open
#3	#1 AND #2
