# Supplementary material for: Minimally invasive versus open radical resection surgery for hilar cholangiocarcinoma: Comparable outcomes associated with advantages of minimal invasiveness
Source: PLoS One. 2021 Mar 11;16(3):e0248534. doi: 10.1371/journal.pone.0248534 (PMC7951922; doi:10.1371/journal.pone.0248534)
Supplement: S2 Table — (PDF) [file pone.0248534.s002.pdf]

Reference	Selection				Comparability	Outcome			Total
	Representativeness of exposed cohort	Selection of the non-exposed cohort	Ascertainment of exposure	Demonstration that outcome of interest was not present at start of study		Assessment of outcome	Adequate follow-up length	Adequacy of follow up	
**Xu et al [31]**	1	1	1	1	2	1	0	1	8
**Zhang et al [32]**	1	1	1	1	2	1	1	1	9
**Jiang et al [33]**	1	1	1	1	2	1	0	1	8
**Zhu et al [34]**	1	1	1	1	1	1	0	1	7
**Gong et al [35]**	1	1	1	1	2	1	0	1	8
**Chou et al [36]**	1	1	1	1	2	1	1	1	9
**Duan et al [37]**	1	1	1	1	2	1	0	1	8
**Chai et al [38]**	1	1	1	1	2	1	1	1	9
**Ratti et al [39]**	1	1	1	1	2	1	1	1	9
